# Use of a wireless ultrasound probe as a portable, noninvasive method for studying reproductive biology in the asp viper, *Vipera aspis*

**DOI:** 10.1002/jez.2608

**Published:** 2022-05-25

**Authors:** Marco Sassoè‐Pognetto, Sonia Acierno, Silvestro Roatta

**Affiliations:** ^1^ Department of Neuroscience “Rita Levi Montalcini” University of Torino Torino Italy

**Keywords:** snake reproduction, *Vipera*, viviparity, wireless ultrasonography

## Abstract

In this study, we investigated the use of wireless ultrasonography as an imaging system to study the reproductive ecology of the asp viper (*Vipera aspis*), a viviparous snake found in southwestern Europe. Female vipers were captured during the summer and immediately scanned to obtain an estimate of the number of embryos. Ultrasound imaging was performed with a pocket‐sized wireless ultrasound probe interfaced with a tablet with a dedicated app. Vipers were then released at the exact capture site after collecting data on body size and weight. We validate wireless ultrasonography as a non‐destructive, effective tool for ultrasonic investigations in the field. Wireless probes are light and compact, which facilitates carriage in rugged terrain. Moreover, the absence of cables simplifies the maneuvers to be made on a small, potentially dangerous snake. Importantly, ultrasound scans can be performed at the capture site, thus minimizing restraint time and handling of gravid females.

## Research Highlights

- We establish wireless ultrasonography as an effective tool for animal fieldwork.
- Wireless ultrasound scanners are light and flexible and can be used in harsh environments where power sources are not accessible.

## Graphical abstract

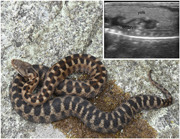

## 1 INTRODUCTION

Reproduction is a crucial aspect of an animal's life‐cycle and one of the main factors determining the fitness and survival of populations. Reptiles are often considered an interesting group for studying reproductive ecology because they provide important insights into the evolution of the amniote egg and of viviparity (Blackburn, 2006; Bonnet et al., 2017; Shine, 1983) as well as on the complex interactions between environmental changes and populations' adaptive responses (see Sparkman et al., 2018 and references therein). Ideally, long‐term studies of wild populations should be carried out to detect intraspecific population differences in reproductive patterns and to examine population dynamics over time (Gilman & Wolf, 2007; Madsen & Shine, 2001; Sparkman et al., 2018; Taylor & DeNardo, 2005). These studies require non‐destructive methods that minimize the impact of experimental manipulations on free‐living animals.

Ultrasonography has been extensively used to investigate reproduction in reptiles, both to diagnose pregnancy in females and to monitor embryonic development (Gartrell et al., 2002; Gilman & Wolf, 2007; Kuchling & Razandrimamilafiniarivo, 1999; Lourdais et al., 2015; Love et al., 1996; Martínez‐Torres et al., 2006; Robeck et al., 1990; Rostal et al., 1990; Sacchi et al., 2012; Sparkman et al., 2018; Stahlschmidt et al., 2011; Taylor & DeNardo, 2005). This imaging method is minimally invasive and, as such, it constitutes an ideal approach for obtaining longitudinal time‐series data from the same populations or even the same individuals (Gilman & Wolf, 2007; Taylor & DeNardo, 2005). However, even after portable ultrasound scanners became available, the use of ultrasonography in the field has been quite limited.

In the last decade, pocket‐sized ultrasound machines have become available (Sicari et al., 2011; Tse et al., 2014). These scanners are light and compact and connect wirelessly to a tablet or smartphone, and have been predicted as the next revolution in medical and veterinary imaging (Woo et al., 2014). Because of their portability, affordability, and ease of use, wireless ultrasound probes have a great potential for field investigations. In this study, we tested the efficacy of a wireless ultrasound system to assess the reproductive status and estimate embryo numbers in the asp (common) viper (*Vipera aspis*, Linnaeus, 1758), a medium‐sized viviparous snake from southwestern Europe (Ursenbacher et al., 2006).

## 2 MATERIALS AND METHODS

**Figure 1:**
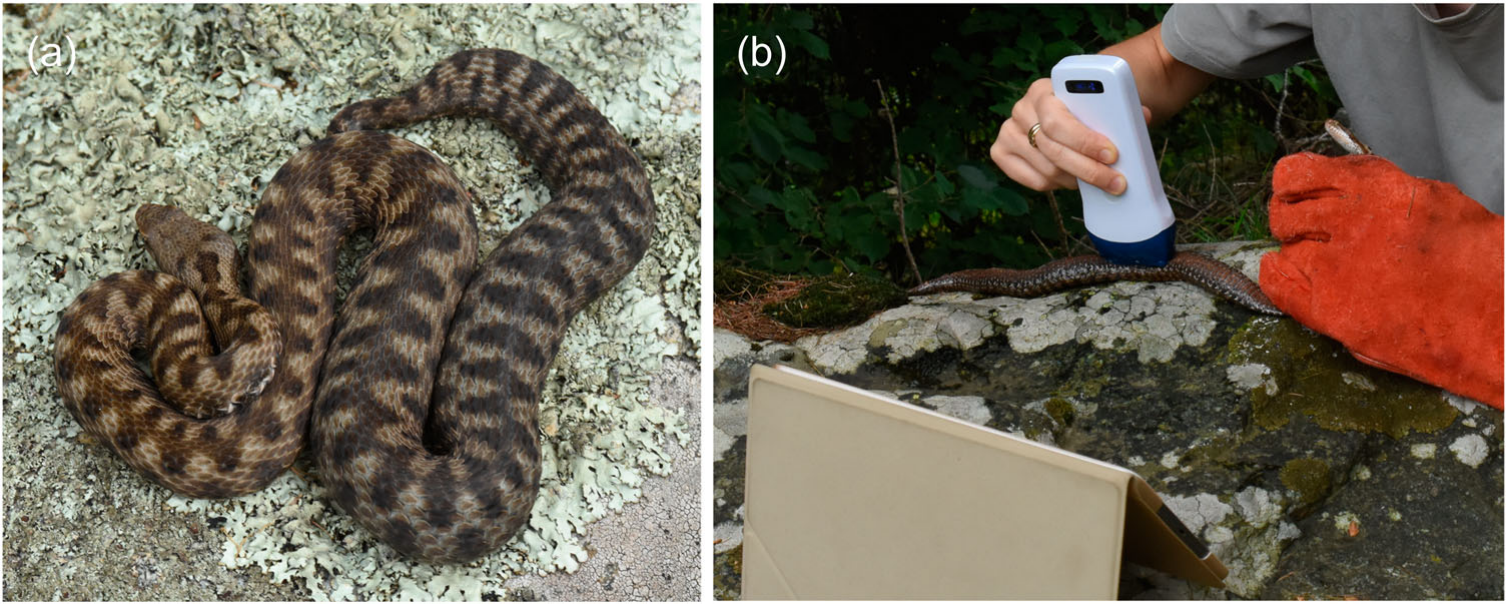
Ultrasound examination of vipers (*Vipera aspis*). (a) One of the gravid females that were investigated. (b) In‐the‐field ultrasound scanning of a gravid viper. Females were examined shortly after capture and then released at the exact capture site.

A total of nine gravid female vipers were captured in the month of August on the Italian Western Alps at altitudes between 1150 and 1515 m asl. Pregnant females are easy to detect due to their increased body mass and characteristic shape of the abdomen (Figure 1a). Snakes were measured (snout‐vent length [SVL] and total length [TL]), weighed, and individually identified by head scale patterns and markings. They were then scanned with a pocket‐sized wireless ultrasound probe (Color Doppler OTE Linear L 102 CD; 7.5–10 MHz) interfaced with a tablet (iPad, 6th generation) with a dedicated app (Wireless USG, Sonostar Technologies). This probe weighs 234 g and its size is 155 × 65 × 28 mm. Each female was scanned ventrally along the rostral‐caudal axis (Figure 1b) and individual “eggs” were counted, while ultrasound images and videos were recorded. The probe was coated with ultrasound gel during the entire procedure. After scanning, the gel was removed with paper towels. All of the procedures were carried out as gently as possible and usually required less than 30 min, after which the vipers were released at the capture sites. *V. aspis* is not listed in Annex IV of EU Habitat Directive and is not protected by local wildlife legislation. No pain was inflicted on the experimental subjects and utmost care was exerted to limit disturbance and restraint time.

## 3 RESULTS

*V. aspis* normally undergoes ovulation in May‐June and gives birth in late August or at the beginning of September, depending on climatic conditions (Lourdais et al., 2004; Luiselli & Zuffi, 2002). We first captured and scanned three vipers in the first half of August (day 8th), then six more about 2 weeks later (August 20th‐25th), thus at relatively advanced stages of gestation. One female was captured and analyzed twice, first on August 8 and then 12 days later (Table 1). All vipers were relatively docile and could be imaged with simple manual restraint (Figure 1b).

**Table 1:** Ultrasonographic estimation of embryo numbers in gravid asp vipers (*Vipera aspis*)

Viper	Date of analysis	SVL (cm)	Body mass (g)	Number of embryos
RIV_16_31	August 8	49.5	106	5
RIV_18_80^a^	August 8	53.4	120	7
RIV_18_80^a^	August 20	nd	111	7
RIV_18_81	August 8	50.6	111	6
ALB_18_08	August 20	55.8	121	5
ALB_18_01	August 24	57	173	9
SOA_18_01	August 25	49	96	6
SOA_18_02	August 25	48.2	104	6
SOA_18_03	August 25	45.7	83	5
SOA_18_04	August 25	46.5	79	5

^a^ Female RIV_18_80 was captured and examined by ultrasonography at two distinct times. Note that the weight of the snake varied by 7.5% between the two imaging sessions.

**Figure 2:**
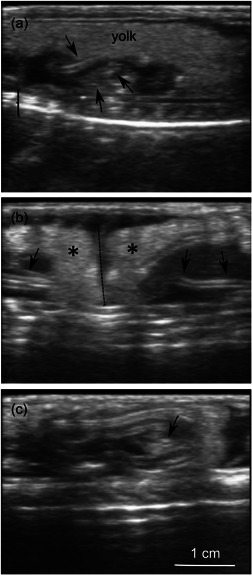
Ultrasound images of individual “eggs” in the oviduct of *Vipera aspis*. In all images, the ventral side is at the top and the vertebral column (bracket) of the female is visible in the dorsal aspect. (a) An individual “egg” with embryonic skeletal elements (arrows) surrounded by a prominent mass of yolk. (b) Two contiguous “eggs” (asterisks) adjoining at the level of the dotted line. Arrows point to echogenic skeletal elements of the embryos. (c) An embryo at a later stage of development. Notice that the amount of yolk has strongly decreased and the embryo occupies most of the egg volume. The arrow likely points to the cranium.

During the first evaluation in early August, ultrasound scanning evidenced elongated “eggs” that were easily discernible due to the accumulation of echogenic yolk material (Figure 2a,b). Embryos with quite distinct hyperechoic skeletal elements were also visible in the dorsal aspect of each “egg.” Heartbeats were clearly discernible in one of the embryos (Movie S1). As gestation progressed, the amount of *vitellus* decreased while embryo size augmented considerably. As visible in Figure 2c, at this later stage of gestation embryos appeared as echogenic vertebral coils occupying most of the egg volume.

The “eggs” were arranged linearly in the caudal half of the snakes. The first “egg” was detected just behind mid‐body, on average at 55.1% ± 5.1 (mean ± SD) of the SVL (N = 7); the last “egg” ended a few centimeters (mean ± SD: 5.7 cm ± 1.07, N = 7) from the cloaca. By scanning vipers longitudinally, we counted individual “eggs.” A video loop (Movie S2) illustrates the imaging of three consecutive “eggs” during a scanning session. A summary of the number of detected embryos, together with female body mass and SVL, is given in Table 1.

We remark that detailed recognition and quantification of individual embryos was easier at the earlier stage that we investigated, due to the higher abundance of yolk revealing individual “eggs” (Figure 2b). However, analysis of the same female in the two imaging sessions resulted in the same count of “eggs.”

## 4 DISCUSSION

Pocket ultrasound transducers allow applications that were previously unthinkable for field researchers. Compared with standard ultrasound machines, they offer improved portability and can be used in remote environments where power sources are not accessible. Being able to perform ultrasound examinations in the field is a major asset, as it minimizes the need for temporary housing of wild animals. Moreover, the absence of cables increases ergonomics and ease of use, simplifying the maneuvers to be made on wild animals, including potentially dangerous ones. While being considerably less expensive than standard ultrasound machines, wireless ultrasound scanners afford good image quality and have been validated for clinical use (e.g., see Jung et al., 2021; Zardi et al., 2019).

In this study, we establish wireless ultrasonography as an effective tool to diagnose pregnancy and estimate litter size in *V. aspis*. Indeed, we detected ovulated eggs with discernible embryos in all females that we judged gravid by visual inspection. Because we released females after ultrasound examination, we could not establish the level of accuracy of our analysis. However, the number of embryos that we counted (Table 1) is compatible with data present in the literature for this species, although litter size presents some variation in distinct populations as it is influenced by climatic conditions and maternal body size (Bonnet et al., 2000; Lourdais et al., 2004; Zuffi et al., 2009). Ultimate verification of the data would have required laboratory housing of females until parturition, which we excluded to minimize disturbance. While the housing of gravid females provides information about offspring and accurate control over ambient parameters (Bonnet et al., 2008; Lourdais et al., 2004, 2015), it potentially affects animals' biology and life‐history data (see e.g., Gilman & Wolf, 2007; Sacchi et al., 2012). Another option would have been the dissection of euthanized individuals, which is difficult to justify given the availability of non‐destructive alternatives. We feel that, once the scanning parameters (probe frequency, placement of the transducer, speed of scanning) have been set properly, it is quite easy to identify individual embryos in snakes, as the “eggs” are relatively large and arranged linearly in the oviduct. Even in lizards, in which many small eggs are clustered in the body cavity, it has been possible to obtain an accurate estimation of clutch size by ultrasonography (Gilman & Wolf, 2007; Sacchi et al., 2012).

We predict that wireless ultrasonography will become a suitable tool for field investigations as well as for diagnostic imaging in reptiles. Different transducers (e.g., linear, convex, micro convex) which operate with different frequencies are available, making it possible to select the most appropriate equipment and imaging configuration for the species of interest. The frequency of transducers can be adjusted to obtain optimal penetration and the best image quality even in larger snakes, such as large boids (Banzato et al., 2013), in turtles (Urbanová & Halán, 2016), and in crocodilians (Lance et al., 2009). Optimal imaging also requires proper identification of the best acoustic window, which might differ in different species. For example, according to Banzato et al. (2012), a dorsolateral approach is preferable for imaging the coelomic cavity in large snakes, as this provides good visualization of most organs while reducing animal distress. Although the present observations were based only on B‐mode (brightness) imaging, the availability of other scanning modes broadens the spectrum of potential applications. For example, color Doppler may be used to reveal blood flow within individual embryonic sacs and thus help in the identification of potential nonviable embryos (Bonnet et al., 2008).

Because of maximal portability and versatility, wireless ultrasonography, which has recently been incorporated in medical and veterinary imaging, promises to become an important tool for animal fieldwork. In the case of reproductive ecology and physiology, potential applications comprise the analysis of longitudinal as well as geographic variation of reproduction dynamics in wild populations.

## CONFLICT OF INTEREST

The authors declare no conflict of interest.

## Supporting information

Supporting information.

Supporting information.

Supporting information.

## Data Availability

The data that support the findings of this study are available from the corresponding author upon reasonable request.
