# Supplementary material for: Use of a wireless ultrasound probe as a portable, noninvasive method for studying reproductive biology in the asp viper, Vipera aspis
Source: J Exp Zool A Ecol Integr Physiol. 2022 May 25;337(7):724–8. doi: 10.1002/jez.2608 (PMC9545915; doi:10.1002/jez.2608)
Supplement: Supplementary file 3 — Supporting information. [file JEZ-337-724-s002.docx]

**Movie 1.** A short video showing heartbeats in a developing viper embryo.

**Movie 2.** Ultrasound examination of a gravid viper (*Vipera aspis*). Three eggs containing embryos come into view sequentially during the rostral-caudal scanning.
